# Complications associated with adolescent childbearing in Sub-Saharan Africa: A systematic literature review and meta-analysis

**DOI:** 10.1371/journal.pone.0204327

**Published:** 2018-09-26

**Authors:** Taran Grønvik, Ingvild Fossgard Sandøy

**Affiliations:** 1 Centre for International Health, University of Bergen, Bergen, Norway; 2 Department of Global Public Health and Primary Care, University of Bergen, Bergen, Norway; 3 Centre for Intervention Science in Maternal and Child Health (CISMAC), Centre for International Health, University of Bergen, Bergen, Norway; TNO, NETHERLANDS

## Abstract

**Objective:**

To examine whether childbearing before age 18 in Sub-Saharan Africa is associated with increased risk of maternal and child complications through a systematic literature review and meta-analysis.

**Methods:**

The literature on adolescent pregnancy and associated complications in Sub-Saharan Africa was reviewed. A systematic electronic database search in Medline and Embase identified relevant papers. Studies were eligible for inclusion if they had numeric data on maternal mortality, pre-eclampsia, eclampsia, preterm birth, low birth weight, small for gestational age, stillbirth, neonatal death or perinatal death. We included studies on adolescents aged 17 years or younger, and with a comparison group of adult women aged between 20 and 35 years. The quality of the articles was assessed. Meta-analyses were conducted when there were at least three included studies with minor clinical heterogeneity in population and outcome measures.

**Results:**

Eighteen studies met our inclusion criteria. There were many studies from Sub-Saharan Africa with data on the age group 15–19 years old, but few studies had separate data on adolescents <18 years old. All included studies were of either moderate or low quality. Adolescents had an increased risk of low birth weight, pre-eclampsia/eclampsia, preterm birth and maternal and perinatal mortality. We found a lower, nonsignificant risk of stillbirth and for small for gestational age babies among the young mothers.

**Conclusion:**

In this systematic review, the findings indicate that young maternal age is associated with some unfavorable outcomes in Sub-Saharan Africa. High quality observational studies that adjust for sociodemographic factors are lacking.

## Introduction

Approximately 16 million adolescents aged 15–19 years old give birth each year in developing regions [1]. Another million girls younger than 15 years old also give birth annually [2]. Adolescent childbearing is associated with health risks for both the mother and the child. In low and middle income countries, complications of pregnancy, childbirth and abortion are estimated to be among the leading causes of death for girls aged 15–19 years old [3]. Higher prevalence among adolescents of hypertensive disorders of pregnancy and higher risk of their babies having low birth weight (LBW) and/or being born prematurely have also been reported from numerous studies from both high and low income countries [4–7].

Whether the association between adolescent pregnancy and adverse outcomes for mothers and newborns is caused by biological immaturity, or is a result of the poorer socioeconomic status of pregnant adolescents, is a topic of some controversy [8, 9]. Adolescent girls are more likely to have unwanted pregnancies, and the girls who become pregnant are often relatively poor, less educated and from rural areas. They are also less likely to access prenatal care, and prenatal care interventions aimed at adolescents have in some studies improved some of the adverse outcomes of adolescent pregnancy [5]. Pregnancy after the age of 16 years in high income countries is not associated with an increased risk of adverse outcomes [8], which further implies that health services are important for the pregnancy outcomes of adolescents.

There are indications that the prevalence of complications such as low birth weight varies considerably between different geographical areas, with the prevalence of LBW being particularly high in South Asia [10]. Because of the geographical variation of complications, we decided to focus on Sub-Saharan Africa in this review since the continent has the highest rates of adolescent pregnancy in the world, with recent estimates of 28% of girls having given birth before the age of 18 in West and Central Africa, and 25% in Eastern and Southern Africa [11]. In addition, recent systematic reviews on complications of adolescent pregnancy have included very few studies from Sub-Saharan Africa [8, 12].

The risk of maternal and newborn complications has been found to be higher for younger compared to older adolescents. However, many studies on adolescent childbearing group all adolescents together, including up to 19 year old girls, even though this could mask the higher risk of adverse outcomes among the younger girls. In this systematic review, studies that included separate measurements of outcomes for adolescents aged 17 years and less were included.

Our objective was to examine whether childbearing before age 18 in Sub-Saharan Africa is associated with increased risk of maternal mortality, preeclampsia/eclampsia, prematurity, low birth weight, small for gestational age babies, stillbirth, perinatal mortality or neonatal mortality compared to adults through a systematic literature review and meta-analysis.

## Methods

### Search strategy and study selection

Systematic electronic database searches in Medline and Embase identified relevant papers. We conducted searches with the thesaurus terms ‘pregnancy in adolescence’ and ‘pregnancy complications’ in Medline and ‘adolescent pregnancy‘ and ‘pregnancy complication’ in Embase, and searches in both databases with the search words listed in Table 1, with restriction to English language articles and citations from 2005 and later. The last search was done on the 1^st^ of December 2017. References cited in systematic review articles on the topic were also reviewed.

**Table 1:** Search words.

Adolescent	Pregnancy	Sub-Saharan Africa	Maternal and child complications
(adolescence or adolescent or adolescents or teenage or teenaged or teen or teens or youth or youths or girl or girls or ((maternal age) and young))	(childbearing or pregnancy or pregnancies or pregnant or childbirth or childbirths or birth or births)	(Angola OR Benin OR Botswana OR Burkina Faso OR Burundi OR Cabo Verde OR Cameroon OR Central African Republic OR Chad OR Comoros OR Congo OR Cote d'Ivoire OR Ivory Coast OR Eritrea OR Ethiopia OR Gabon OR Gambia OR Ghana OR Guinea OR Guinea-Bissau OR Kenya OR Lesotho OR Liberia OR Madagascar OR Malawi OR Mali OR Mauritania OR Mauritius OR Mozambique OR Namibia OR Niger OR Nigeria OR Rwanda OR (Sao Tome) OR Principe OR Senegal OR Seychelles OR Sierra Leone OR Somalia OR South Africa OR South Sudan OR Sudan OR Swaziland OR Tanzania OR Togo OR Uganda OR Zambia OR Zimbabwe OR Africa)	((maternal mortality) or (maternal death) or (maternal deaths) or (pre-eclampsia) or preeclampsia or eclampsia or preterm or premature or prematurity or (low birthweight) or (low birth weight) or (small for gestational age) or (still birth) or stillbirth or stillbirths or (neonatal death) or (neonatal deaths) or (perinatal death) or (perinatal deaths) or (newborn death) or (newborn deaths) or (neonatal mortality))

All returned papers were exported into Endnote X7, and after a duplication check they were screened to see if they were eligible for inclusion. Both authors conducted the systematic search and independently screened all of the returned papers. The first stage involved screening of titles and abstracts to see if the articles appeared to be relevant. The papers that were considered to potentially be eligible for inclusion, were kept for screening of full texts. When there was uncertainty regarding whether a paper was relevant or not, the full text was always screened. When the full text of an article was not available in the electronic databases and journals that the University of Bergen subscribes to, a librarian was consulted to find it. Reasons for excluding papers after fulltext screening were recorded. Disagreements or questions regarding whether a paper was eligible for inclusion, were discussed among the authors to achieve consensus.

### Eligibility criteria

Studies were eligible for inclusion if they had numeric data from Sub-Saharan Africa on any of the following outcomes of interest: maternal mortality, pre-eclampsia, eclampsia, preterm birth, low birth weight, small for gestational age, stillbirth, neonatal death and perinatal death. We included English language publications from 2005 or later, with a clearly defined sample of adolescents aged 17 years or younger from the general population, and with a comparison group of adult women aged between 20 and 35 years old.

Commentaries, editorials, case reports, general reviews, qualitative studies and conference abstracts were not eligible for inclusion. We excluded studies that did not present separate findings from Sub-Saharan Africa. Studies focusing on special groups such as patients with HIV were also excluded, as these were not considered to have a study population that was representative of the general population. Lack of an older control group (within the set age limits) and age data not being properly presented were other exclusion criteria.

### Data extraction, quality assessment and qualitative synthesis

Data from the included studies was extracted using a data extraction form. From each study, data was collected on design, setting and time, population, comparison group, inclusion and exclusion criteria, baseline imbalances, outcomes and definitions, sample size, withdrawals, exclusions and missing data, results for each outcome of interest, confounding factors accounted for, self-reported limitations of the study, funding sources and possible conflicts of interests.

Critical appraisal of the included studies was conducted using Quality assessment tool for Observational Cohort and Cross-Sectional Studies [13] and the Newcastle-Ottawa Scale (NOS) [14]. These tools were used to assess the risk of bias in the individual studies, and to grade the quality of the study as strong, moderate or low. The risk of bias in the selection of the study population, the comparability between the study groups, and the valid and consistent ascertainment of the exposure and outcome of interest were some of the quality parameters that were assessed with these tools. Both the data extraction and the quality assessment were conducted independently by both authors, and then compared and discussed to reach consensus.

The qualitative synthesis included a description of the characteristics, and findings of the included studies based on the data extraction findings. Effect estimates with confidence intervals for each of the included studies were reported, and homogeneity in the estimates and whether conducting a meta-analysis would be appropriate was assessed for each of the outcomes.

### Statistical analysis

Odds ratios (OR) with 95% confidence intervals for the different outcomes in each study were calculated. Meta-analysis was conducted when there were at least three included studies with minor clinical heterogeneity in population and outcome measures. Statistical heterogeneity was assessed using the Chi^2^ test, the Tau^2^ and the I^2^ statistic. We conducted random-effects meta-analysis to allow for expected differences in results among studies, with the Mantel-Haenzel method for weighting to calculate a summary OR and a forest plot to present the results for the different outcomes. A result was considered to be statistically significant when the 95% confidence interval of the OR excluded 1. Meta-analysis was performed using Review Manager 5.3.

## Results

### Study selection

**Fig 1:**
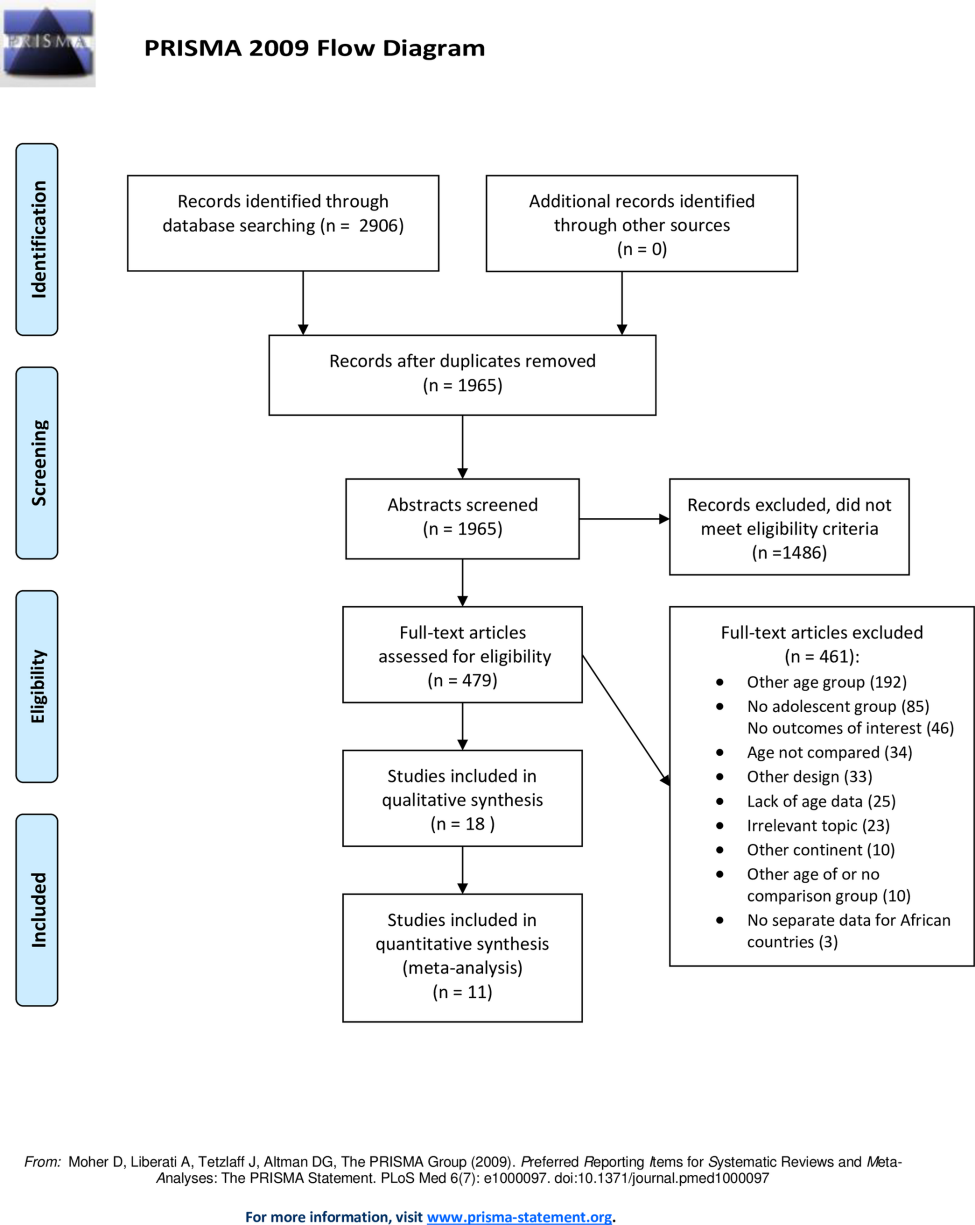
Literature review flow diagram.

Eighteen studies met our inclusion criteria, out of 1965 potentially relevant publications found from the database search. Screening of reference lists did not result in any additional relevant studies. After title and abstract screening, 1486 studies were excluded, while full text screening was done for 479 articles. There were many studies from Sub-Saharan Africa with data on the age group 15–19 years old, but few studies had separate data on adolescents <18 years old. There was one study we were not able to retrieve full text of [15]. Exclusions at each stage with reasons are presented in Fig 1. For most of the excluded articles, more than one exclusion criteria applied, and for these the first detected reason is the one reported in the flow diagram.

### Included studies

The eighteen included studies were primarily hospital or health clinic based patient record reviews, except for two studies which were based on household surveys [16, 17], one which was based on public death notification forms [18], and one which used various data sources [19]. Two of the studies, both by Mombo-Ngoma et al., reported on some of the same data [20, 21], and to avoid adding the same data twice, only one of the studies was included in the meta-analysis.

The studies had varying age groups of adolescents. Three studies had data on adolescents aged less than 15 years [16, 18, 19], four studies had an adolescent group aged 15 years or less [22–25], five studies included adolescents aged 16 years or less [20, 21, 26–28], while six included adolescents up to 17 years old [17, 29–34]. There were three studies from Cameroon [23, 27, 28], two each from Nigeria[25, 29], Tanzania [24, 32], South Africa [18, 34] and Zambia [16, 17], one study each from Namibia [31], Gabon [20], Ghana [33], Ethiopia [19] and Sudan [26], and two multi-country studies: Ganchimeg et al. with data from Algeria, Angola, Democratic Republic of Congo, Niger, Nigeria, Kenya and Uganda [22], and Mombo-Ngoma et al. which included data from Benin, Gabon and Mozambique [21]. See Table 2 for study characteristics, and Tables 3 and 4 for results for the individual studies.

**Table 2:** Characteristics of included studies.

Study	Study type	Setting and time	Ages of adolescents *Ages of comparison group*	Inclusion and exclusion criteria	Outcomes and definitions
Ujah et al. 2005	Patient record review	Jos University Teaching Hospital, Nigeria Jan 1985-Dec 2001	≤15 *20–24*	Not reported	Maternal deaths; women who died during pregnancy or childbirth in the maternity ward of the hospital
Wort et al. 2006	Cross-sectional patient record review	Kilosa Hospital, Tanzania June 2001- October 2002	≤15, n = 34 *≥20*, *n = 281*	Inclusion: vaginal births Exclusions: Twin births, stillbirths, cesarean section. Unclear whether triplets excluded.	Low birth weight, <2500 g
Van Dillen et al. 2008	Retrospective patient record review	Onandjokwe Lutheran Hospital, Namibia February 2002 –August 2002	14–17, n = 76 *≥20*, *n = 371*	Inclusion: Primiparous women, singleton births Exclusion: Twin births, unknown age of mother	Low birth weight, <2500 g Perinatal mortality; all deaths occurring after 28 weeks gestation with weight greater or equal to 1000 g Maternal mortality Stillbirth (macerated)
Adam et al. 2009	Cross-sectional descriptive study	Khartoum teaching hospital, Sudan October 2007 –January 2008	≤16, n = 29 *20–24*, *n = 203*	Inclusion: Primiparous women, singleton births Exclusion: > 24 years old	Preterm birth, <37 weeks of gestation Low birth weight, <2500 g
Nkwabong et al. 2009	Retrospective patient record review	Yaoundé Teaching Hospital, Cameroon January 2004 –December 2004	≤15, n = 11 16, n = 13 17, n = 21 *20–25*, *n = 403*	Inclusion: Nulliparous teenagers or women aged 20–25 years old	Early neonatal death
Adeyinka et al. 2010	Retrospective case-control study, patient record review	The University College Hospital, Ibadan, Nigeria January 2007 –November 2008	<18, n = 45 *20–35*, *n = 90*	Inclusion: Adolescents aged <18 years old or adults aged between 20 and 35	Pre-eclampsia* Eclampsia** Low birth weight, <2500 g Stillbirths
Zeck et al. 2010	Retrospective patient record review	The Kilimanjaro Christian Medical Centre, Moshi, Tanzania.	<18, n = 209 *22–27*, *n = 1341*	Inclusion: Primiparas Within the set age categories	Intrauterine fetal death LBW, <2500 g at term Preeclampsia at the time of delivery
Tebeu et al. 2011	Case-control study, patient record review	Maroua Regional Hospital, Cameroon June 2005 –May 2007	13–16, n = 53 *20–34*, *n = 330*	Inclusion: Cases had hypertensive disorders in pregnancy Exclusion: Twin gestations, chronic hypertension	Pre-eclampsia/eclampsia***
Ganchimeg et al. 2013	Secondary analysis using facility-based cross-sectional patient record data from the WHO Global Survey on Maternal and Perinatal Health	Health institutions in 24 countries in Africa, Latin America and Asia. Africa: Algeria, Angola, Democratic republic of Congo, Niger, Nigeria, Kenya and Uganda. 2004–2005 in Africa	Africa: ≤15, n = 551 *20–24*, *n = 10242*	Inclusion: Health institutions with ≥1000 deliveries per year and capable of performing caesarean sections. Nulliparous women ≤24 years of age Singleton neonate of BW ≥500 g or gestational age ≥22 weeks if BW was missing	Low birthweight, <2500 g Preterm birth; births <37 completed weeks of gestation. Perinatal death**** Maternal deaths; intra-hospital deaths occurring on or before the 8^th^ day postpartum
Lukonga et al. 2015	Cross-sectional population based data	National household survey Zambia Demographic and Health survey (ZDHS) 2007	12–17, n = 2649 (# of births) *25–29*, *n = 233* (# of births)	Inclusion: Women who gave birth to live infants within 5 years preceeding the survey Available and complete records of the babies for the first 28 days Exclusion: Incomplete or missing records for the baby	Neonatal death, up to 28 days postpartum
Banda et al. 2015	Cross-sectional data	Zambia. Data from national population census 2010,	10–14 20–24		Pregnancy-related deaths: Death of a woman while pregnant or within 42 days of termination of pregnancy
Ibrahim et al. 2015	Retrospective patient record review. Purposive sampling method from birth registry folders	Tamale Teaching Hospital, Northern Ghana, data collected from 2000 and 2010	<18, n = 12 25–34, n = 761		Low birth weight, <2500 g
De Wet et al. 2016	Secondary data analysis of Death Notification Forms (DNF)	South Africa. DNFs and household surveys from 2006–2012.	10–14 *20–24*	Inclusion: Completed forms where the deceased was female and pregnancy status was confirmed	Maternal death: Direct maternal causes of death while pregnant Pregnancy-related deaths: All deaths while pregnant
Njim et al. 2016	Retrospective patient record review	Bueau Regional Hospital, South-West region of Cameroon Jan. 2007 –Dec. 2012	≤16, n = 78 *≥20*, *n = 4450*	Inclusion: Complete records of women who gave birth at the hospital Exclusion: Gestational age <28 completed weeks, multiple gestations, incomplete information	Preterm delivery Low birth weight
Mombo-Ngoma et al. 2016	Prospective multinational cohort study	Data from a RCT (MiPPAD) in Benin, Gabon, Mozambique and Tanzania Sept 2009 –Dec.2013	≤16, n = 248 *20–30*, *n = 2376*	Inclusion: HIV negative women, gestational age (GA) <28 weeks at first ANC visit, willing to participate and give birth at the study health facility Exclusion: Allergy to any of the study drugs. Any other ongoing serious condition. Stillbirths, multiple gestations. *Tanzania data on PTD was excluded in the paper because of a systematic error in the assessment of GA*	Low birth weight, <2500 g Preterm delivey, <37 completed weeks of gestation, estimated at ANC visit with bimanual palpation, and at delivery by the Ballard score
Moodley et al. 2016	Cross-sectional patient record review	A regional hospital in Durban, South Africa. July–Dec. 2011 and Jan.–June 2014	<18, n = 827 *25–34*, *n = 3662*	Inclusion: Women with viable pregnancies delivering neonate >_500 g with recorded birth outcomes Exclusion: Multiple births	Premature births, <37 completed weeks of gestation Low birth weight, <2500 g Stillbirth Small for gestational age (gestational age at delivery based on mothers last normal menstrual period, or ultrasound, or a combination of both)
Mombo-Ngoma et al. 2017	Analysis of two prospective cohort studies (MiPPAD and IDEA)	Two health institutions in Lambaréne and Fougamou, Gabon Sept 2009 –Nov. 2013	≤16, n = 66 *20–30*, *n =* 587	Inclusion: Participation in MiPPAD or IDEA trials Exclusion: Missing delivery data, miscarriage or stillbirth, multiple gestations	Low birth weight, <2500 g
Tessema et al. 2017	Secondary data from Global burden of Disease study from 2013.	Ethiopia, 1990–2013. Various data sources; sibling stories, censuses, maternal mortality surveillance and verbal autopsy analysis.	10–14 20–24 25–29		Maternal death: Death of a woman while pregnant or within 42 days of termination of pregnancy

* Hypertension (140/90 mmHg on two occasions 4h apart) and proteinuria (0,3g/dl) in the second half of pregnancy

** Associated with convulsions, oliguria (4400ml/24h), increased tendon reflex, pain in the right hypochondriac region

*** Women with a diastolic blood pressure of at least 90 mmHg or a systolic BP of at least 140 mmHg were considered to have hypertensive disorder in pregnancy

**** Perinatal deaths included fresh and macerated stillbirths and early neonatal deaths, defined as the inta-hospital death of a liveborn neonate during the first 7 days after delivery or earlier if the discharge occurred before 7 days

**Table 3:** Adolescent pregnancy and adverse outcomes for the baby.

Study	Age groups	LBW	Preterm birth	Perinatal death	Neonatal death	Stillbirths	Small for gestational age	Adjusted ORs
Wort 2006	≤15, n = 34 *≥20*, *n = 281*	<16: 29% >19: 18.9% OR = 1.79 (0.81, 3.97)	-	-	-	-	-	-
Van Dillen 2008	14–17, n = 76 *≥20*, *n = 371*	14–17, n = 76 *≥20*, *n = 371*	-	-	Early neonatal death: 0/76 vs 4/371	1/76 vs 6/371 OR = 0.81 (0.096, 6.84)	-	-
Adam 2009	≤16, n = 29 *20–24*, *n = 203*	7/29 (24.1%) vs 23/203 (11.3%) OR = 2.49 (0.96, 6.47)	2/29 (6.8%) vs 29/203 (14.2%) OR = 0.44 (0.10, 1.97)	-	-	-	-	-
Nkwabong 2009	≤15, n = 11 16, n = 13 17, n = 21 *20–25*, *n = 403*	-	-	-	Early neonatal death: OR = 29.6 (4.4, 199.5)	-	-	-
Adeyinka 2010	<18, n = 45 *20–35*, *n = 90*	22.2% vs 20.22%, p = 0.635 OR = 1.14 (0.48, 2.73)	-	-	-	24.4% vs. 21.1% OR = 1.21 (0.52, 2.82)	-	-
Zeck 2010	<18, n = 209 22–27, n = 1341	26 (12.4%) vs 94 (7%) OR = 1.89 (1.19, 2.99)	-	-	-	1.9% vs 1.2% OR = 1.62 (0.54, 4.88)	-	-
Ganchimeg 2013	Africa: ≤15, n = 551 20–24, n = 10242	19.7% vs 9.6% OR = 2.32 (1.86, 2.89)	21.8% vs 11% OR = 2.25 (1.82, 2.79)	7.6% vs 4.5% OR = 1.75 (1.26, 2.43)	-	-	-	ORs not adjusted for Africa separately
Lukonnga 2015	12–17, n = 2649 (# of births) *25–29*, *n = 233* (# of births)	-	-	-	Neonatal deaths: 25–29 vs 12–17: OR = 1.61 (0.91, 2.87)	*-*	-	Neonatal deaths: aOR = 0.83 (0.29, 2.35) 25–29 years vs. 12–17 years
Ibrahim 2015	<18, n = 12 25–34, n = 761	<18 ref. OR = 0.60 (0.17, 2.79)*	-	-	-	-	-	LBW: aOR = 0.65 (0.17, 2.41) <18 ref.
Njim 2016	≤16, n = 78 *≥20*, *n = 4450*	OR = 2.26 (1.29, 3.94)	OR = 2.45 (1.40, 4.28)					
Mombo-Ngoma 2016	≤16, n = 248 *20–30*, *n = 2376*	OR = 1.96 (1.35, 2.83)	OR = 1.82 (1.09, 3.03)	-	-	-	-	LBW: 1) aOR = 2.06 (1.37, 3.12) 2) aOR final model = 1.29 (0.81, 2.06) PTD: 1) aOR = 1.73 (1.01, 2.98) 2) aOR final model = 2.16 (1.10, 4.24)
Moodley 2016	<18, n = 827 *25–34*, *n = 3662*	<18 ref. OR = 0.93 (0.75, 1.17)	<18 ref. OR = 0.85 (0.71, 1.02)	-	-	<18 ref. OR = 1.8 (1.05, 3.1)	<18 ref. OR = 0.86 (0.66, 1.13)	<18 ref. LBW: aOR = 0.84 (0.66, 1.06) PTD: aOR = 0.78 (0.64, 0.94) Stillbirth: aOR = 1.86 (1.06, 3.25) SGA: aOR = 0.81 (0.61, 1.07)
Mombo-Ngoma 2017	≤16, n = 66 *20–30*, *n =* 587	OR = 1.27 (0.61, 2.63)						LBW: aOR = 1.66 (0.68, 4.02)

* Ibrahim et al. reported an unadjusted OR of 0.80 (0.62, 1.05) for 25–34 year olds compared to <18 year olds.

Our calculations gave a different unadjusted OR of 0.60, which is the one reported in Table 4. However, in the meta-analysis we used their adjusted OR of 0.65 (OR = 1.54 when 25–34 year olds is the reference group).

**Table 4:** Adolescent pregnancy and adverse outcomes for the mother.

Study	Age groups	Pre-eclampsia/ eclampsia	Maternal mortality	Adjusted ORs
Adeyinka 2010	<18, n = 45 20–35, n = 90	Eclampsia: 9/45 (20%) vs 3/90 (3.33%) OR = 7.25 (1.86, 28.33)	-	-
Banda 2015	10–14 20–24	-	Pregnancy-related mortality rate, 10–14: 9338/100.000 20–24: 557/100.000	-
De Wet 2016	10–14 20–24	-	Probability of dying during pregnancy, 10–14: 0.0001 20–24: 0.0039	-
Ganchimeg 2013	Africa: ≤15, n = 551 20–24, n = 10242	-	73.1/10,000 births vs 19.6/10,000 births	ORs not adjusted for Africa separately
Tebeu 2011	13–16, n = 53 20–34, n = 330	25/53 vs 76/330 OR = 2.98 (1.64, 5.42)	-	-
Ujah 2005	≤15 20–24	-	573/100,000 total deliveries vs. approx. 500/100,000 total deliveries	-
Zeck 2010	<18, n = 209 22–27, n = 1341	Pre-eclampsia: 11/209 (5.3%) vs 20/1341 (1.5%) OR = 3.67 (1.73, 7.77)	-	-
Tessema 2017	10–14 20–24 25–29	-	2013: 10–14: ≈120/100,000 live births 20–24: ≈140/100,000 live births 25–29: ≈85/100,000 live births	-

### Risk of bias

All studies were of either moderate or low quality. This was due to lack of adjustment for potential confounding factors and various other limitations in the individual studies, including the risk of selection bias when conducting hospital-based studies. Five of the studies [17, 20, 21, 33, 34] had adjusted for sociodemographic characteristics. Ganchimeg et al. [22] was a multi-country study, and adjusted for sociodemographic factors in the pooled analysis, but did not adjust the separate analysis of data from African countries. See Table 5 for a summary of the quality assessment.

**Table 5:** Summary of quality assessment results.

Study	Clearly stated research question/ objective?	Clearly specified and defined study population?	Subjects from same populations? Inclusion criteria prespecified and applied uniformly?	Sample size justification, power description, or variance and effect estimates provided?	Outcome measures clearly defined, valid, reliable and implemented consistently?	Key potential confounding variables measured and adjusted for?	Representativeness of the exposed cohort	Assessment of outcome
Ujah	Yes	Yes	Yes	No	Not reported	No	Somewhat representative	Record linkage
Wort	Somewhat	Yes	Yes	No	Yes	No	No description of the derivation of the cohort	Record linkage
Van Dillen	Somewhat	Yes	Yes	No	LBW and perinatal mortality defined. Stillbirths not defined.	No	Somewhat representative	Record linkage
Adam	Yes	Yes	Probably, but not explicitly stated	Effect estimates, but no sample size justification	Yes	No	Somewhat representative	Self report. Probably also medical records, not explicitly stated
Nkwabong	No	No	Not mentioned	Effect estimates, but no sample size justification	No	No	No decription of the derivation of the cohort	Record linkage
Adeyinka	Yes	No	Not reported	No	Stillbirth not defined, eclampsia and LBW defined	No	Not description of the derivation of the cohort	Record linkage
Zeck	Somewhat	Yes	Somewhat. Inclusion/exlusion not described well	No	Probably, not clearly mentioned	No	Somewhat representative	Record linkage
Tebeu	Yes	No–controls not properly described	Not mentioned	Effect estimates, but no sample size justification	Yes	Some risk factors have adjusted ORs, but not age	No description of the derivation of the cohort	Record linkage
Ganchimeg	Yes	Yes	Yes	Effect estimates, but no sample size justification	Yes	Yes, but not for Africa separately	Somewhat representative	Record linkage
Lukonga	Somewhat	No	Yes	No	Partly, not a clear definition	Yes	Truly representative	Self report and record linkage
Banda	Yes	Yes	Yes	No	Clearly defined, but not reliably measured because of dependency on recall of household members	No	Truly representative	Self report (reported by househould member)
De Wet	Yes	Yes	Yes	No	Yes	No	Truly representative	Record linkage
Ibrahim	Yes	Yes	Yes	Yes	Yes	Some. Not adjusted for education or wealth	No description of the derivation of the cohort	Record linkage
Mombo-Ngoma 2016	Yes	Yes	Yes	Yes	Yes	Yes	Somewhat representative	Record linkage
Mombo-Ngoma 2017	Yes	Yes	Participants from two cohort studies	Yes	Yes	Some. Not adjusted for education or wealth	No description of the derivation of the cohort	Record linkage
Moodley	Yes	Yes	Yes	Yes	For most outcomes, no definition found for stillbirth	Some. Not adjusted for education or wealth	No description of the derivation of the cohort	Record linkage
Njim	Yes	Yes	Yes	No	No	No	No description of the derivation of the cohort	Record linkage
Tessema	Yes	Not clearly stated in this paper	Not specified	No	Yes	No	Somewhat representative	Record linkage

### Low birth weight

Eleven of the included studies had data on LBW, defined as birth weight less than 2500 g [20–22, 24, 26, 28, 29, 31–34]. Ten of them reported a higher incidence among the adolescent group [20–22, 24, 26, 28, 29, 32–34]. For four of the studies [21, 22, 28, 32], the difference was significant. These were also four of the five studies with the largest sample sizes. However, after controlling for BMI, parity, literacy, plasmodium infection and middle upper arm circumference, Mombo-Ngoma et al. found a nonsignificant association (aOR = 1.29 (0.81, 2.06)) between young maternal age and LBW [21]. When controlling for country, trimester of first antenatal care visit, group of preventive malaria treatment and infant gender instead, the association was significant.

We performed a meta-analysis for this outcome, including ten of the studies. Mombo-Ngoma et al. from 2017 [20] was not included in the meta-analysis, as it mostly reported on the same data as the other included Mombo-Ngoma article from 2016. The 2016 article had a larger sample size and was a multi-country study, and hence was preferred for inclusion in the meta-analysis. Mombo-Ngoma 2017 assessed urogenital schistosomiasis and adverse outcomes in Gabon, based on data from two prospective cohort studies. They found a nonsignificant increased risk of LBW among adolescents, aOR = 1.66 (0.68, 4.02).

**Fig 2:**
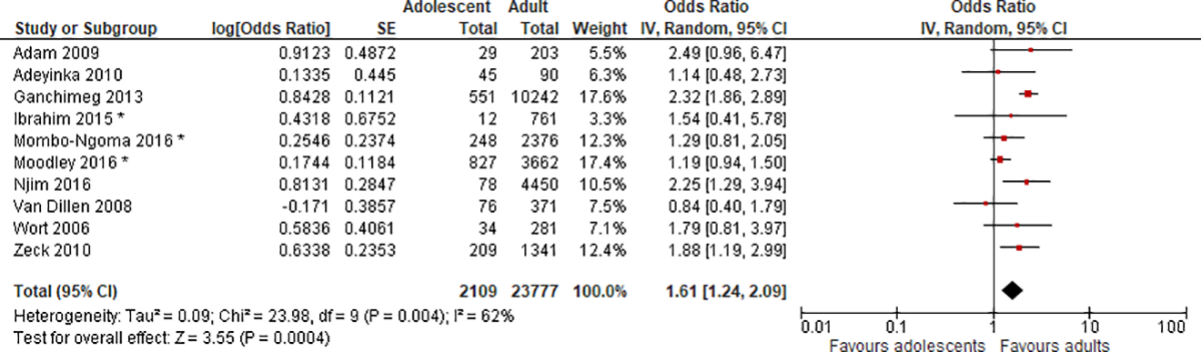
Forest plot for low birth weight. * Adjusted ORs. IV, inverse variance; CI, confidence interval.

The summary OR for the meta-analysis was 1.61 (1.24, 2.09) (Fig 2). The I^2^ statistic is 62%, which suggests moderate heterogeneity.

### Preterm birth

Five of the included studies had data on preterm birth, defined as birth before 37 completed weeks of gestation. For Ganchimeg et al., gestational age (GA) at birth was defined as “the number of completed weeks of gestation based on the estimated delivery date in the clinical records” [22]. Moodley et al. based GA on the mother’s last normal menstrual period or by ultrasound [34], and Mombo-Ngoma et al. estimated GA at the mother’s first antenatal care visit with bimanual palpation, and at delivery using the Ballard score [21]. Adam et al. and Njim et al. did not report how they assessed gestational age [26, 28].

Four of the studies found a significant difference between the adolescent and the adult group, with a higher risk of preterm birth for the adolescents [21, 22, 28, 34]. Adam et al. did not find a significant difference, and the incidence of preterm birth was higher in the comparison group of adults. However, the sample size of adolescents in this study was very small (n = 29) resulting in an imprecise estimate [26]. Two of the studies controlled for potential confounders, Moodley et al. and Mombo-Ngoma et al., and both found that the association between young maternal age and preterm birth was significant [21, 34]. Moodley et al. found a nonsignificant association before adjustment, OR = 1.18 (0.98, 1.41), but the association was significant after controlling for mode of delivery, pregnancy term category and HIV status, aOR = 1.28 (1.06, 1.55).

**Fig 3:**
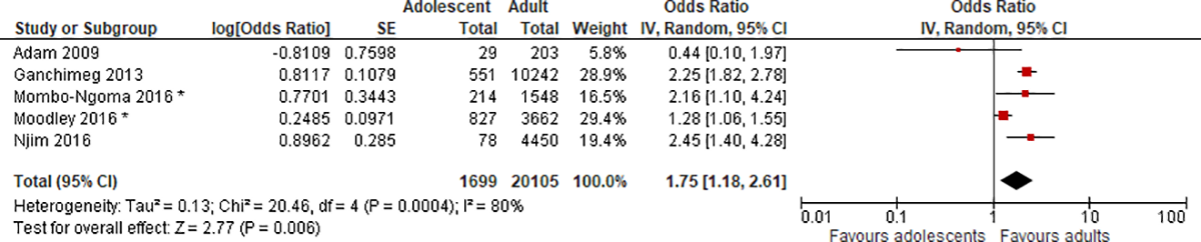
Forest plot for preterm birth. * Adjusted ORs. IV, inverse variance; CI, confidence interval.

A meta-analysis was conducted with the five studies, and the summary OR was 1.75 (1.18, 2.61) (Fig 3). The I^2^ statistic is 80%, which suggests important heterogeneity.

### Stillbirth

**Fig 4:**
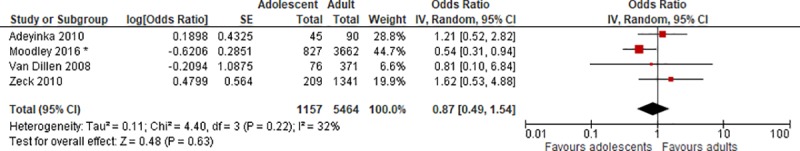
Forest plot for stillbirth. * Adjusted OR. IV, inverse variance; CI, confidence interval.

Four of the included studies had data on stillbirth. Van Dillen included only macerated stillbirths [31]. The other studies did not report what definition they used for stillbirth. Adeyinka et al. found a nonsignificantly higher risk of stillbirth among the adolescent group [29]. Zeck et al. reported a significantly higher rate of intrauterine fetal deaths among adolescents, p <0.01, analyzed by Student’s paired t-test [32]. However our calculations indicate that the crude OR was nonsignificant, OR = 1.62 (0.53, 4.88). Van Dillen et al. and Moodley et al. on the other hand found a higher incidence of stillbirth among the adult group [31, 34]. Only Moodley et al. found a significant association [34]. For Van Dillen et al., the age of the mother was unknown for two macerated stillbirths. Even though the definitions of stillbirth were not reported for all of the studies, and there was some missing data for one of the studies [31], we conducted a meta-analysis, and found a summary OR = 0.87 (0.49, 1.54) (Fig 4). The I-squared statistic is 32%, which indicates low to moderate study heterogeneity for this outcome.

### Perinatal and neonatal deaths

Ganchimeg et al. included fresh and macerated stillbirths and early neonatal deaths in their definition of perinatal mortality. Early neonatal death was defined as “the intra-hospital death of a liveborn neonate during the first 7 days after delivery or earlier if the discharge occurred before 7 days”. They found a higher incidence of perinatal mortality in the adolescent group, OR = 1.75 (1.26, 2.43) [22].

Two studies had data on early neonatal deaths, but none of them reported their definition of this outcome. Nkwabong et al. found a much higher risk in the adolescent group of girls ≤15 years old compared to 20–25 year olds, OR = 29.6. They reported a 95% confidence interval from 27.9 to 31.2 although our calculations indicate a much wider 95% confidence interval from 4.4 to 199.5. The odds ratio was 6.20 (1.01, 38.14) for girls ≤17 years old [23]. Van Dillen et al. found no early neonatal deaths among the newborns of adolescent mothers [31].

Lukonga et al. was the only included study that had data on neonatal deaths up to 28 days postpartum. The adult group aged 25–29 years old had a lower risk of neonatal death than the adolescent group aged 12–17 years old. The difference was not significant (aOR = 0.83 (0.29, 2.35)) [17]. The study also compared younger adolescents to the age group 18–24 years old, and found that this age group had a significantly lower risk of neonatal death than the 12–17 year old group (aOR = 0.47 (0.23, 0.96)).

### Small for gestational age

Only one study included data on small for gestational age (SGA) infants. Moodley et al. [34] found that women aged 25–34 years old had a lower risk of having a SGA infant than adolescent mothers under 18 years of age. The difference was not significant, aOR = 0.81 (0.61, 1.07).

### Pre-eclampsia/eclampsia

Three of the included studies had results for pre-eclampsia and eclampsia. Adeyinka et al defined pre-eclampsia as hypertension (≥140/90 mmHg on two occasions 4 hours apart) and proteinuria (>0,3g/dl) in the second half of pregnancy, while eclampsia was defined as “associated with convulsions, oliguria (>400ml/24h), increased tendon reflex, pain in the right hypochondriac region” [29]. Tebeu et al. also stated that women were considered to have a hypertensive disorder of pregnancy when they had a systolic blood pressure of at least 140 mmHg or a diastolic blood pressure of at least 90 mmHg. Women who had chronic hypertension were excluded, however the article does not describe if and how they differentiated between gestational hypertension and pre-eclampsia. No description of proteinuria is given, however the results are presented as women who had pre-eclampsia/eclampsia [27]. Zeck et al. reported pre-eclampsia at the time of delivery, based on hypertension, proteinuria and resting edema [32].

**Fig 5:**
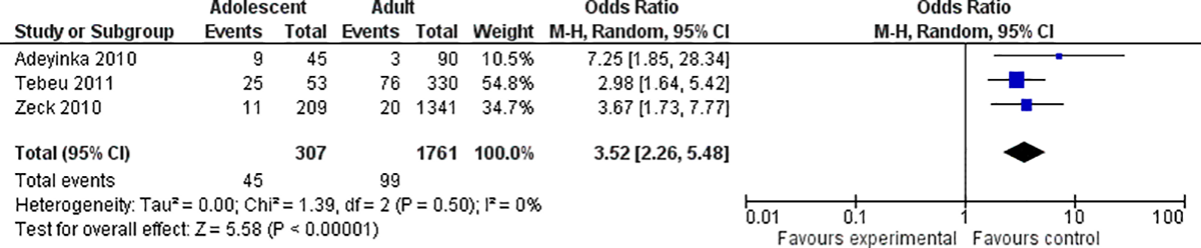
Forest plot for pre-eclampsia/eclampsia. IV, inverse variance; CI, confidence interval.

All the three studies found a significant difference between the adolescent and the adult group, with a higher incidence in the adolescent group. Adeyinka et al [29] found that 20% in the adolescent group had pre-eclampsia or eclampsia, compared to 3% in the comparison group. Tebeu et al. found a greater risk for the age group 13–16 years old compared to the age group 20–34 years old (odds ratio 2.974, (1.627, 5.427) [27]. Zeck et al found 5% of pre-eclampsia in the adolescent group, compared to 1.5% in the adult group, and the difference was significant, OR = 3.665 (1.671, 7.723) [32]. Even though there are some differences and uncertainty concerning the definitions for this outcome, we considered the studies to be similar enough to conduct a meta-analysis, which gave a summary OR = 3.52 (2.26, 5.48) (Fig 5). The I^2^ statistic is 0%, which shows no observed heterogeneity.

### Maternal mortality

Five studies reported data on maternal mortality. Ujah et al. [25] studied all women who died during pregnancy or childbirth in the maternity ward of the hospital, but did not define the outcome further, while Ganchimeg et al. defined maternal mortality as “intra-hospital deaths occurring on or before the eighth day postpartum” [22]. Ujah et al. analyzed data from a hospital during a 17 year period in Nigeria, and found a maternal mortality ratio (MMR) of 573/100,000 total deliveries for the adolescent group aged 15 and less. The result for the comparison group aged 20–24 years old is only reported in a graph in the article, which shows a MMR of approximately 500/100,000 total births [25]. Ganchimeg et al. found a maternal mortality ratio of 731/100,000 births among the adolescents from Sub-Saharan Africa in their study, while the ratio for adults was 196/100,000 births [22].

Tessema et al. used secondary data from the Global burden of Disease study from 2013 [35], which explored several different data sources on maternal mortality in Ethiopia from 1990 to 2013. They found that the age groups of 10–14 and 20–24 were the groups with the highest maternal mortality ratios throughout this period. In 1990 and 1995, girls aged 10–14 had the highest maternal mortality ratio. Later, the 20–24 year olds had higher ratios. In 2013, the MMR for 10–14 year olds was approximately 120/100,000 live births, while it was 140/100,000 live births for 20–24 year olds. In comparison, the 25–29 year old group had a MMR of approximately 85/100,000 live births.

Banda et al. analysed data from the national population census in Zambia from 2010. They reported pregnancy-related deaths, defined as deaths occurring while pregnant, during childbirth, or in a 6 week period after giving birth. Based on their findings, the pregnancy-related mortality ratio was 9338/100.000 live births for girls aged 12–14 year old, compared to 557/100.000 live births for 20–24 year olds [16].

De Wet et al. analyzed Death Notification Forms to examine maternal and pregnancy-related deaths, but they did not study deaths that occurred in the postnatal period. They calculated the probability of dying during pregnancy, and found that the probability was just 0.01% for young adolescents aged 10–14, compared to 0.39% for women aged 20–24. Excluding indirect causes of death during pregnancy, the risk of dying of direct maternal causes for 10–14 year olds was 0.007%, and for 20–24 year olds 0.39%. The main causes of death for adolescents in their study were hypertension, abortion and injuries [18]. This was the only study that reported a lower risk of mortality for adolescents. They reported a high number of missing cases, up to 32.44% [18]. Three other studies with data on these outcomes, found that adolescents had a higher risk of pregnancy-related and maternal mortality [16, 22, 25]. Tessema et al. found a MMR for 10–14 year olds that was lower than the 20–24 year olds for recent years, while it was higher than the MMR for 25–29 year olds [19].

Because of the differing ways these four studies collected and reported data on maternal mortality and pregnancy-related deaths, we considered them too heterogeneous in population and outcome measures, and therefore did not conduct a meta-analysis of this outcome.

## Discussion

### Summary of evidence

The results of this review indicate that there is an association between young maternal age and low birth weight, preterm birth, pre-eclampsia/eclampsia and maternal and perinatal mortality in Sub-Saharan Africa. We found a lower (but non-significant) risk of stillbirth and for small for gestational age baby among the young mothers. High quality observational studies that adjust for sociodemographic factors are lacking.

#### Limitations of included studies

A major limitation of the included studies in this review, is that most of them did not adjust for sociodemographic factors. Adolescent birth rates are higher in rural areas, and among adolescents who are less educated or from poor households [11]. Poor pregnancy outcomes are also associated with many factors such as poverty, low education and lack of prenatal care, and when none of these possible confounding factors are adjusted for, the higher risk of adverse pregnancy outcomes of adolescents could be at least partly due to their poorer socioeconomic status rather than their age. Among the studies included in this review, only Ganchimeg et al. and Lukonga et al. attempted to adjust for socioeconomic status by including educational attainment of the mother in the multiple logistic regression models. However, Ganchimeg et al. did not have separate adjusted analyses for African countries. None of the included studies adjusted for wealth, employment, income, urban or rural residence or tribe/ethnic group. As most of the included studies were retrospective record reviews, the reason for not adjusting for socioeconomic differences could be lack of information on education status, wealth and other socioeconomic characteristics of the study participants.

High quality studies from Sub-Saharan Africa were lacking. A systematic literature review and meta-analysis by Gibbs et al. from 2012 included only studies of at least moderate quality that adjusted for sociodemographic confounders, and those studies were primarily from high income countries [12]. In our review, some higher quality studies which included data from Sub-Saharan Africa were excluded because the data was pooled with data from other parts of the world [7, 36, 37] or because the adolescent group consisted of girls up to 19 years old, or the comparison group included girls 18–19 years old [38] [7]. There is a need for more high quality studies that distinguish between younger and older adolescents, instead of grouping them all together, in order to come up with firm conclusions on the differences in risks for mothers who are younger than 18 years compared to adults.

The systematic review conducted by Gibbs et al. concluded that it appears that there “may be a true biological effect of very young age at first pregnancy (<15 years or so) on infant health, through the increased risk of preterm birth and LBW”. The findings on LBW in Gibbs’ review are comparable to those in this review. Gibbs et al. grouped adolescents aged 16 years and younger into different age stratums to look at LBW. They got a summary OR from the meta-analysis of 1.42 (1.06, 1.89) for the oldest age stratum aged 14–16 years old, and 1.82 (1.60, 2.07) for the youngest age stratum aged 10–14 years old. In this review, our summary OR for adolescents aged 17 years or younger was 1.61 (1.24, 2.09).

Fall et al. pooled data from five different birth cohorts from South Africa, Brazil, Guatemala, India and the Philippines, to examine associations between maternal age and birth outcomes, as well as adult outcomes for the babies. They found that having an adolescent mother (<19 years old) was associated with LBW, preterm birth, small for gestational age babies, 2-year stunting, and lower completion of secondary school [39]. These associations remained after adjustment for socioeconomic status, height and parity. Fall et al. later presented separate data on the different ages of adolescent mothers [40]. They concluded that the children born to mothers aged 15–16 years old, were especially disadvantaged compared to those born to older mothers aged 19 and older. The association with lower birthweight for the babies of the adolescent mothers was still significant after adjusting for socioeconomic factors, but not after additionally adjusting for parity.

The meta-analysis by Gibbs et al. and the study by Fall et al. show that the negative effect young maternal age has on low birth weight and preterm birth is likely to persist even when adjusting for socioeconomic factors, especially for the younger adolescents. Thus it seems likely that the results found in our review would still show a disadvantage for adolescent mothers and their babies, even if all the studies had adequately adjusted for socioeconomic factors. Gibbs et al. only had one included study with pre-eclampsia as an outcome, but that study also found a higher risk for adolescents (aOR = 5.4 (1.2, 25) [41].

In this review, the one included study that studied perinatal mortality, Ganchimeg et al., found a significantly increased risk of perinatal death for the babies of adolescents girls [22], while the pooled data from the studies on stillbirth resulted in a reduced, but nonsignificant, risk of stillbirth for adolescents compared to adults. A recent multi-country study from low and middle income countries by Althabe et al. found similar results, with a reduced, nonsignificant association for stillbirth when adolescents <15 years old were compared to adults (RR = 0.80 (0.38, 1.71)), and an increased, but also nonsignificant, risk of perinatal mortality among <15 year olds (RR = 1.19 (0.67, 2.11)) [37].

Another limitation with these studies is the small sample sizes of some of the studies, which is associated with a risk of random variations in the number of cases with complications, possibly influencing the overall associations. In addition, all except for three [16–18] are hospital-based or health clinic-based studies. This means that the samples may not be representative of the general population, as births outside of health facilities are common in Sub-Saharan Africa, particularly in rural areas [42, 43]. The proportion of institutional deliveries varies between countries, but in many African countries less than half of deliveries are at a health facility [44]. The fact that the included studies are from referral hospitals and clinics implies that there will be a higher proportion of complicated pregnancies among women delivering there, which could result in an overestimation of risks. In addition, adolescents in Sub-Saharan Africa are more likely to give birth outside of hospitals or health clinics than adult women [45], and adolescent pregnancies are more common in rural areas. This means that when the data in this review is mostly from hospital-based studies in urban settings, the adverse outcomes of childbearing in this group may be underestimated. Some of the inclusion criteria in the studies may also limit their external validity. For example, Mombo-Ngoma et al. only included HIV negative pregnant women, and reports this as one possible limitation [21].

Differences in the definition and measurements of adverse pregnancy outcomes, or lacking information about definitions used, further complicated the comparison of studies in this review. For low birth weight, all studies had the same outcome definition. However, the studies that assessed neonatal mortality used different definitions, partly due to different follow-up times. The common definition of early neonatal death is death within the first 7 days of life, but the duration of follow-up in all the hospital-based studies was short, i.e. only until discharge from the hospital. Most women stay shorter than 7 days in the hospital after delivery, and thus the medical records may not have captured all deaths that occurred within the first week. Similarly, Ganchimeg et al., who report data on perinatal mortality (which per definition refers to stillbirths and mortality during the first 7 days of life), were not able to follow up women who were released from the health institutions before 7 days had passed [22]. This means that the incidence of perinatal mortality might have been greater than reported in their study.

Assessment of gestational age was performed with varying methods in the different studies. None of them had access to ultrasound for all their estimations, and several studies listed lack of information on gestational age [33] or inaccurate estimations [34] as a limitation. This could lead to misclassification of premature and full-term babies. If accurate estimations of gestational age had been available and routinely used, we probably would have found more studies that had assessed small for gestational age as an outcome, and could differentiate the babies with low birth weight as either premature or small for gestational age.

Information bias such as recall bias is not likely to be a major problem in studies based on medical records, but can be a challenge in household surveys where respondents are asked to recall events in the past. Lukonga et al. [17] and Banda et al. [16] both used data from household surveys, but since the outcomes of interest from these studies are neonatal death and pregnancy-related deaths, dramatic events that are not likely to be forgotten, recall bias was not likely to have been big concerns in these studies either. However, incomplete medical records were more likely to be a problem in the included studies. The completeness of the records is likely to be suboptimal in settings with high patient loads and limited numbers of health care professionals. Missing data was reported in some of the studies, but not mentioned in others. Van Dillen et al. had 7 cases of unknown age of the mother, including for two macerated stillbirths [31]. In a study like this with few outcome events, inclusion of two more cases in the analysis could have relatively big effects on the odds ratio, and this could also have affected the summary OR for stillbirth. Wort et al. reported missing data on nine births [24]. Missing information on pregnancy status could be a problem in both Banda [16] et al. and de Wet et al. [18]. De Wet et al. reported that missing cases for pregnancy status was up to 32.44% during their study period [18].

### Limitations of the review process

This systematic review is limited by the decision to focus on English language articles and only to include studies from 2005 and later. In addition, a limitation is that we only searched two databases. However, the two we chose are the biggest biomedical databases, and by also screening the reference lists of other review articles, it appears likely that we have found most of the relevant articles on the studied outcomes. We were able to retrieve full text of all articles that we considered might be relevant, except for one [15].

Since there is controversy regarding the topic of adolescent pregnancy and the risk of adverse pregnancy outcomes, negative results might be likely to be published, but there is still a possibility that publication bias had an impact on the results of this review and meta-analyses.

## Conclusions

The findings of this review indicate that there is an association between young maternal age and low birth weight, pre-eclampsia/eclampsia, preterm birth and maternal and perinatal mortality in Sub-Saharan Africa. There were no significant associations for stillbirth or for small for gestational age.

Most of the included studies did not adjust for key potential confounding variables.

This review identifies specific gaps in the literature on pregnancy among adolescents less than 18 years old in Sub-Saharan Africa. There were few studies on younger adolescents, as most studies grouped all adolescents up to 19 years old together. High quality observational studies on young adolescent pregnancy, adjusted for sociodemographic factors, are lacking. Future research addressing adolescent pregnancy in Sub-Saharan Africa should use several age subgroups for adolescents, and adjust for all important confounders.

## Supporting information

S1 Table PRISMA checklist. (DOCX)

## References

[1] WHO. Adolescent pregnancy. Updated January 2018. Available from: http://www.who.int/mediacentre/factsheets/fs364/en/.

[2] Neal S, Matthews Z, Frost M, Fogstad H, Camacho AV, Laski L. Childbearing in adolescents aged 12–15 years in low resource countries: a neglected issue. New estimates from demographic and household surveys in 42 countries. Acta Obstetricia et Gynecologica Scandinavica. 2012 9;91(9):1114–8. 10.1111/j.1600-0412.2012.01467.x . English. 22620274

[3] Patton GC, Coffey C, Sawyer SM, Viner RM, Haller DM, Bose K, et al Global patterns of mortality in young people: a systematic analysis of population health data. The Lancet.374(9693):881–92. 10.1016/S0140-6736(09)60741-8 19748397

[4] Paranjothy S, Broughton H, Adappa R, Fone D. Teenage pregnancy: Who suffers? Archives of Disease in Childhood. 2009 3;94(3):239–45. 10.1136/adc.2007.115915 . 19019886

[5] Scholl TO, Hediger ML, Belsky DH. Prenatal care and maternal health during adolescent pregnancy: A review and meta-analysis. Journal of Adolescent Health. 1994;15(6):444–56. . 7811676 10.1016/1054-139x(94)90491-k

[6] Olausson PMO, Cnattingius S, Goldenberg RL. Determinants of poor pregnancy outcomes among teenagers in Sweden. Obstetrics and Gynecology. 1997 3;89(3):451–7. . 9052604 10.1016/s0029-7844(97)00009-4

[7] Fall CHD, Sachdev HS, Osmond C, Restrepo-Mendez MC, Victora C, Martorell R, et al Association between maternal age at childbirth and child and adult outcomes in the offspring: a prospective study in five low-income and middle-income countries (COHORTS collaboration). The Lancet Global Health. 2015 7//;3(7):e366–e77. 10.1016/S2214-109X(15)00038-8 25999096 PMC4547329

[8] Cunnington AJ. What's so bad about teenage pregnancy? Journal of Family Planning & Reproductive Health Care. 2001;27(1):36–41. . 12457546 10.1783/147118901101194877

[9] Stevens-Simon C, Beach RK, McGregor JA. Does incomplete growth and development predispose teenagers to preterm delivery? A template for research. Journal of Perinatology. 2002;22(4):315–23. 10.1038/sj.jp.7210694 . 12032796

[10] UNICEF W. Low Birthweight: Country, regional and global estimates UNICEF, New York, 2004.

[11] UNFPA. Adolescent pregnancy: A Review of the Evidence Population and Development Branch Technical Division. UNFPA, New York, 2013.

[12] Gibbs CM, Wendt A, Peters S, Hogue CJ. The impact of early age at first childbirth on maternal and infant health. Paediatric and Perinatal Epidemiology. 2012;26 Suppl 1:259–84. 10.1111/j.1365-3016.2012.01290.x . 22742615 PMC4562289

[13] NIH. Quality Assessment Tool for Observational Cohort and Cross-Sectional Studies. Available from: http://www.nhlbi.nih.gov/health-pro/guidelines/in-develop/cardiovascular-risk-reduction/tools/cohort.

[14] Wells GA, Shea, B., O'Connel, D., Peterson J, Welch V, Losos M et al. The Newcastle-Ottawa Scale (NOS) for assessing the quality of nonrandomised studies in meta-analyses. Available from: http://www.ohri.ca/programs/clinical_epidemiology/oxford.asp.

[15] Tiemo SJ, Wannang NN, Sariem CN, Auta A, Omale S. Pharmacological intervention of pre-eclampsia and eclampsia: A case study of a tertiary health institution in jos, Nigeria. Drug Invention Today. 2012 4;4(4):360–2. . English.

[16] Banda R, Sandoy IF, Fylkesnes K, Janssen F. Impact of pregnancy-related deaths on female life expectancy in Zambia: Application of life table techniques to census data. PLoS ONE. 2015 29 10;10 (10) (e0141689). 10.1371/journal.pone.0141689 . 26513160 PMC4626102

[17] Lukonnga E, Michelo C. Factors associated with neonatal mortality in the general population: Evidence from the 2007 zambia demographic and health survey (zdhs); a cross sectional study. Pan African Medical Journal. 2015;20(13). . 10.11604/pamj.2015.20.64.5616 PMC4450022 26090022

[18] de Wet N. Pregnancy and death: An examination of pregnancy-related deaths among adolescents in South Africa. SAJCH South African Journal of Child Health. 2016 9;10(3):151–5. .

[19] Tessema GA, Laurence CO, Melaku YA, Misganaw A, Woldie SA, Hiruye A, et al Trends and causes of maternal mortality in Ethiopia during 1990–2013: findings from the Global Burden of Diseases study 2013. BMC public health. 2017 02 2;17(1):160 10.1186/s12889-017-4071-8 . 28152987 PMC5290608

[20] Mombo-Ngoma G, Honkpehedji J, Basra A, Mackanga JR, Zoleko RM, Zinsou J, et al Urogenital schistosomiasis during pregnancy is associated with low birth weight delivery: analysis of a prospective cohort of pregnant women and their offspring in Gabon. International Journal for Parasitology. 2017 01 1;47(1):69–74. 10.1016/j.ijpara.2016.11.001 . 28003151

[21] Mombo-Ngoma G, Mackanga JR, Gonzalez R, Ouedraogo S, Kakolwa MA, Manego RZ, et al Young adolescent girls are at high risk for adverse pregnancy outcomes in sub-Saharan Africa: An observational multicountry study. BMJ Open. 2016 01 6;6 (6) (no pagination)(011783). . 10.1136/bmjopen-2016-011783 PMC4932321 27357200

[22] Ganchimeg T, Mori R, Ota E, Koyanagi A, Gilmour S, Shibuya K, et al Maternal and perinatal outcomes among nulliparous adolescents in low- and middle-income countries: a multi-country study. BJOG: An International Journal of Obstetrics & Gynaecology. 2013;120(13):1622–30; discussion 30. 10.1111/1471-0528.12391 . 23924217

[23] Nkwabong E, Fomulu JN. Adolescent pregnancies and deliveries: Problems encountered. Tropical Doctor. 2009 1;39(1):9–11. 10.1258/td.2008.080047 . 19211412

[24] Uddenfeldt Wort U, Warsame M, Brabin B. Birth outcomes in adolescent pregnancy in an area with intense malaria transmission in Tanzania. Acta Obstetricia et Gynecologica Scandinavica. 2006 01 7;85(8):949–54. 10.1080/00016340600756870 . 16862473

[25] Ujah IA, Aisien OA, Mutihir JT, Vanderjagt DJ, Glew RH, Uguru VE. Factors contributing to maternal mortality in north-central Nigeria: a seventeen-year review. African journal of reproductive health. 2005 12;9(3):27–40. . 16623187

[26] Adam GK, Elhassan EM, Ahmed AM, Adam I. Maternal and perinatal outcome in teenage pregnancies in Sudan. International Journal of Gynaecology & Obstetrics. 2009;105(2):170–1. 10.1016/j.ijgo.2008.11.028 . 19116177

[27] Tebeu PM, Foumane P, Mbu R, Fosso G, Biyaga PT, Fomulu JN. Risk factors for hypertensive disorders in pregnancy: A report from the Maroua Regional Hospital, Cameroon. Journal of Reproduction and Infertility. 2011;12(3):227–34. . 23926507 PMC3719289

[28] Njim T, Choukem SP, Atashili J, Mbu R. Adolescent Deliveries in a Secondary-Level Care Hospital of Cameroon: A Retrospective Analysis of the Prevalence, 6-Year Trend, and Adverse Outcomes. Journal of Pediatric and Adolescent Gynecology. 2016 01 12;29(6):632–4. 10.1016/j.jpag.2016.05.011 27262835

[29] Adeyinka DA, Oladimeji O, Adekanbi TI, Adeyinka FE, Falope Y, Aimakhu C. Outcome of adolescent pregnancies in southwestern Nigeria: a case-control study. Journal of Maternal-Fetal & Neonatal Medicine. 2010;23(8):785–9. 10.3109/14767050903572166 . 20082596

[30] Anim JT. Pregnancy related causes of deaths in Ghana: a 5-year retrospective study. Ghana Med J. 2013 01 12;47(4):158–63. . 24669020 PMC3961851

[31] van Dillen J, van Beijeren E, van Roosmalen J. Perinatal outcome of primiparous teenagers in northern Namibia. Tropical Doctor. 2008;38(2):122–5. 10.1258/td.2007.070093 . 18453514

[32] Zeck W, Wilkinson J, Obure J, Masenga G, Ulrich D, Oneko O. Comparison of obstetrical risk in adolescent primiparas at tertiary referral centres in Tanzania and Austria. Journal of Maternal-Fetal & Neonatal Medicine. 2010;23(12):1470–4. 10.3109/14767051003678077 . 21067304

[33] Ibrahim A, O'Keefe AM, Hawkins A, Hossain MB. Levels and determinants of low birth weight in infants delivered under the national health insurance scheme in Northern Ghana. Maternal and child health journal. 2015 01 6;19(6):1230–6. 10.1007/s10995-014-1628-3 . 25355049

[34] Moodley T, Moodley D, Sebitloane M, Maharaj N, Sartorius B. Improved pregnancy outcomes with increasing antiretroviral coverage in South Africa. BMC Pregnancy & Childbirth. 2016;16:35 10.1186/s12884-016-0821-3 . 26867536 PMC4750240

[35] Global Burden of Disease Study 2013 (GBD 2013) Results by Location, Cause, and Risk Factor: Seattle Institute for Health Seattle Institute for Health Metrics and Evaluation (IHME). Available from: http://www.healthdata.org/gbd.

[36] Ganchimeg T, Ota E, Morisaki N, Laopaiboon M, Lumbiganon P, Zhang J, et al Pregnancy and childbirth outcomes among adolescent mothers: a World Health Organization multicountry study. BJOG: An International Journal of Obstetrics & Gynaecology. 2014;121 Suppl 1:40–8. 10.1111/1471-0528.12630 . 24641534

[37] Althabe F, Moore JL, Gibbons L, Berrueta M, Goudar SS, Chomba E, et al Adverse maternal and perinatal outcomes in adolescent pregnancies: The Global Network's Maternal Newborn Health Registry study. Reproductive Health. 2015;12 Suppl 2:S8 10.1186/1742-4755-12-S2-S8 . 26063350 PMC4464033

[38] Kujala S, Waiswa P, Kadobera D, Akuze J, Pariyo G, Hanson C. Trends and risk factors of stillbirths and neonatal deaths in Eastern Uganda (1982–2011): a cross-sectional, population-based study. Tropical Medicine and International Health. 2017 01 1;22(1):63–73. 10.1111/tmi.12807 . 27910181

[39] Fall CH, Sachdev HS, Osmond C, Restrepo-Mendez MC, Victora C, Martorell R, et al Association between maternal age at childbirth and child and adult outcomes in the offspring: a prospective study in five low-income and middle-income countries (COHORTS collaboration). The Lancet Global Health. 2015;3(7):e366–77. 10.1016/S2214-109X(15)00038-8 . Pubmed Central PMCID: EMS67899. 25999096 PMC4547329

[40] Fall CH, Osmond C, Haazen DS, Sachdev HS, Victora C, Martorell R, et al Disadvantages of having an adolescent mother. The Lancet Global Health. 2016;4(11):e787–e8. 10.1016/S2214-109X(16)30263-7 . 27765286 PMC5722194

[41] Phupong V, Suebnukarn K. Obstetric outcomes in nulliparous young adolescents. The Southeast Asian journal of tropical medicine and public health. 2007 1;38(1):141–5. . Epub 2007/06/02. eng. 17539260

[42] Moyer CA, Mustafa A. Drivers and deterrents of facility delivery in sub-Saharan Africa: a systematic review. Reproductive Health. 2013. PubMed PMID: PMC3751820. 10.1186/1742-4755-10-40 PMC3751820 23962135

[43] Diamond-Smith N, Sudhinaraset M. Drivers of facility deliveries in Africa and Asia: regional analyses using the demographic and health surveys. Reproductive Health. 2015 03/04/received 01/08/accepted;12:6 PubMed PMID: PMC4320522. 10.1186/1742-4755-12-6 25595063 PMC4320522

[44] Wang W, Alva S, Wang S, Fort A. Levels and trends in the use of maternal health services in developing countries Calverton, Maryland, USA: ICF Macro, 2011.

[45] Magadi MA, Agwanda AO, Obare FO. A comparative analysis of the use of maternal health services between teenagers and older mothers in sub-Saharan Africa: Evidence from Demographic and Health Surveys (DHS). Social Science & Medicine. 2007 3//;64(6):1311–25. 17174017 10.1016/j.socscimed.2006.11.004

